# Resistance Training Induces Improvements in Range of Motion: A Systematic Review and Meta-Analysis

**DOI:** 10.1007/s40279-022-01804-x

**Published:** 2023-01-09

**Authors:** Shahab Alizadeh, Abdolhamid Daneshjoo, Ali Zahiri, Saman Hadjizadeh Anvar, Reza Goudini, Jared P. Hicks, Andreas Konrad, David George Behm

**Affiliations:** 1 School of Human Kinetics and Recreation, Memorial University of Newfoundland, St. John’s Newfoundland and Labrador, St. John’s, NL A1C 5S7 Canada; 2 Department of Sport Injuries, Physical Education and Sport Sciences Faculty, Shahid Bahonar University, Kerman, Iran; 3 Institute of Human Movement Science, Sport and Health, Graz University, Graz, Austria; 4 Technical University of Munich, Munich, Germany

## Abstract

**Background:**

Although it is known that resistance training can be as effective as stretch training to increase joint range of motion, to date no comprehensive meta-analysis has investigated the effects of resistance training on range of motion with all its potential affecting variables.

**Objective:**

The objective of this systematic review with meta-analysis was to evaluate the effect of chronic resistance training on range of motion compared either to a control condition or stretch training or to a combination of resistance training and stretch training to stretch training, while assessing moderating variables.

**Design:**

For the main analysis, a random-effect meta-analysis was used and for the subgroup analysis a mixed-effect model was implemented. Whilst subgroup analyses included sex and participants’ activity levels, meta-regression included age, frequency, and duration of resistance training.

**Data Sources:**

Following the systematic search in four databases (PubMed, Scopus, SPORTDiscus, and Web of Science) and reference lists, 55 studies were found to be eligible.

**Eligibility Criteria:**

Controlled or randomized controlled trials that separately compared the training effects of resistance training exercises with either a control group, stretching group, or combined stretch and resistance training group on range of motion in healthy participants.

**Results:**

Resistance training increased range of motion (effect size [ES] = 0.73; *p* < 0.001) with the exception of no significant range of motion improvement with resistance training using only body mass. There were no significant differences between resistance training versus stretch training (ES = 0.08; *p* = 0.79) or between resistance training and stretch training versus stretch training alone (ES = − 0.001; *p* = 0.99). Although “trained or active people” increased range of motion (ES = 0.43; *p* < 0.001) “untrained and sedentary” individuals had significantly (*p* = 0.005) higher magnitude range of motion changes (ES = 1.042; *p* < 0.001). There were no detected differences between sex and contraction type. Meta-regression showed no effect of age, training duration, or frequency.

**Conclusions:**

As resistance training with external loads can improve range of motion, stretching prior to or after resistance training may not be necessary to enhance flexibility.

**Supplementary Information:**

The online version contains supplementary material available at 10.1007/s40279-022-01804-x.

## Key Points

**Table:** 

Resistance training with external loads can improve range of motion to a moderate magnitude
Improvements in range of motion are not significantly different between resistance training and stretch training
Additional stretching prior to or after resistance training may not be necessary to enhance flexibility
Stretch training can still be advocated as a fitness and training component for much of the population and included as a component of a warm-up prior to competition

## Introduction

Stretching was long considered an essential component of warm-ups, fitness, and health [1]. However, the use of stretching as a warm-up component or training routine (i.e., stretching over several weeks) to improve range of motion (ROM), performance, and health has been subjected to a number of counter arguments over the past 20+ years [1–7]. For example, it has been ubiquitously reported since the late 1990s that incorporating static stretching as a pre-activity strategy can lead to performance (e.g., strength, power, speed, balance) impairments [1, 3, 4, 6, 8]. However, a number of reviews [1, 3, 4, 6, 8] have demonstrated that the study designs used in many of these studies lacked ecological validity. Hence, critical analyses of the literature were necessary to demonstrate that if static stretching of less than 60 s per muscle group is incorporated into a warm-up involving dynamic activities, the possibility of significant performance impairments is trivial [1, 3, 4, 6, 8]. However, there is little controversy regarding the effectiveness of acute and chronic (training) stretching for the improvement of joint ROM in healthy populations [1, 9–11]. However, stretch training may not be the only technique for improving ROM and thus it is important that other recommendations for improving ROM, such as resistance training (RT), also be critically evaluated.

Recent commentaries [2, 5] have suggested that possible benefits of stretch training such as improvements in flexibility, balance, cardiovascular measures, alleviation of pain, and decreased injury incidence among others can either be provided by other training modalities (e.g., RT) or stretching is not the most effective activity to provide such benefits (e.g., decreased injury incidence). Nuzzo [5] suggested in his Current Opinion article that chronic RT induced similar increases in ROM as stretch training. Recently, a meta-analysis [12] evaluated 11 studies and reported a non-significant difference between stretch training and RT with a small effect size (ES) in favor of stretching (Hedges’g = − 0.22; *p* = 0.21). However, neither the commentaries nor the meta-analysis evaluated the specific type of RT (i.e., free weights vs machine RT, vs Pilates, vs calisthenics) effects on ROM. Considering the concept of RT specificity (e.g., angle and task specificity), [13, 14] it might be expected that RT with a restricted ROM such as calisthenics or some machine-based RT might not provide similar flexibility improvements as with free weight training through a full ROM.

Typical RT movements involve concentric, isometric, and eccentric muscle contractions. As eccentric contractions can provide higher resistive forces or loads [15, 16] of a lengthening muscle–tendon unit, eccentric contractions may provide a greater stimulus for increasing ROM. There are several reports of substantial ROM increases following eccentric RT [17–19]. More clarity is necessary to ascertain whether there are significant differences in ROM with the different types of contractions.

While many studies do highlight that chronic RT in general can increase ROM [20–26], further in-depth analysis is needed to validate whether the improvement is relatively similar between RT and stretch training. Furthermore, any significant effects of RT on ROM may be moderated by variables such as the sex and trained state of the individual or the frequency and duration [27, 28] of RT. If RT in general or specific types of RT can provide similar improvements in ROM as reported in prior stretching studies, then additional stretching exercises may be removed from the typical training session. Moreover, it is not known whether there is an additive effect of combining RT and stretch training on ROM. Hence, the objective of this research was to conduct a comprehensive systematic review with a meta-analysis to evaluate the effect of chronic RT on ROM compared to controls, stretch training, as well as any possible additive ROM effects of RT and stretch training, with consideration of moderating variables such as the type, frequency, and duration of RT, as well as participants’ sex, age, and activity level (i.e., trained state).

## Methods

This systematic review with meta-analysis was conducted according to the suggestions from Moher and colleagues and meets the Preferred Reporting Items for Systematic reviews and Meta-Analyses (PRISMA) guidelines [29].

### Search Strategy

A literature search following PRISMA review guidelines was performed by six of the co-authors in pairs of two using PubMed, SPORTDiscus, Web of Science, Scopus, and Google Scholar databases. After identifying the eligible studies, data were extracted by two co-authors and if consensus was not reached then a third author provided additional advice. The topic was systematically searched in February 2022 using a Boolean search strategy with the operators “AND”, “OR”, and a combination of the following title keywords: resistance exercise, resistance training, strength, strength training, endurance, endurance training, range of motion, flexibility, stretch, stretching. For example, the following query was used in PubMed database: [“resistance exercise” OR “resistance training” OR strength OR “strength training” OR endurance OR “endurance training” AND “range of motion” OR flexibility OR stretch OR stretching].

Based on our knowledge of the area, we also contributed additional studies from our own computer libraries. Furthermore, we conducted searches of our personal computer databases for related articles and conducted additional ‘snowballing’ searches throughout the process of conducting the review and analysis, which located some newer studies not available when we conducted the initial systematic search. The search ended in May 2022.

### Inclusion and Exclusion Criteria

This review included studies that separately compared the training effects of RT exercises with a control group, stretching group, or combined stretch and RT group on ROM in healthy participants. We included controlled and randomized controlled trials written in English with a longitudinal training design (i.e., pre- to post-training comparison). Moreover, we excluded studies that investigated the combined effects of RT with other treatments such as aerobic training. We further excluded conference papers or theses.

### Extraction of the Data

From all the included papers, the characteristics of the participants (i.e., sex, trained state, age), sample size number, study design, characteristics of the intervention (frequency and duration of RT, exercise type), muscles tested by the ROM test, and the pre- and post-intervention values plus standard deviation of the main variable ROM were extracted. If the full paper did not provide all the data required for the meta-analysis, the corresponding authors were contacted via e-mail and Research Gate.

### Statistics and Data Synthesis

The meta-analysis was conducted using the Comprehensive Meta-Analysis software according to the suggestions of Borenstein et al. [30]. Consequently, a random-effect meta-analysis was used to assess the ES (standardized mean difference) for the ROM effects. If any study reported more than one ES, as suggested by Borenstein et al. the mean of all the outcomes (ESs) within one study was used for the analysis and defined with the term “combined” [30]. Although there is no general rule of thumb [30], we only performed a meta-analysis when three or more studies could be included in the respective analysis. Moreover, to assess possible relations in the moderating variables, we conducted a meta-regression (i.e., age of the participants, weeks of intervention, and training sessions per week). Additionally, by using a mixed-effect model, we conducted various subgroup analyses with the activity level of the participants (untrained and sedentary vs trained and active), sex (male vs female vs mixed), type of contraction (eccentric, concentric, and isometric), type of exercise (body weight vs free weight vs machine vs Pilates vs resistance bands vs mixed), and the joint tested (elbow vs hip vs knee vs shoulder vs trunk vs trunk and hip). Trained and active individuals were defined as people who regularly participated in exercise and sports on a weekly basis. Q-statistics were applied [30] to determine if there were differences between the ESs of the subgroups. Hopkins suggested to define the standardized mean difference of < 0.2, 0.2–0.6, 0.6–1.2, 1.2–2.0, 2.0–4.0, and > 4.0 as trivial, small, moderate, large, very large, and extremely large, respectively [31]. To assess the heterogeneity, I^2^ statistics were calculated among the ESs, and thresholds of 25%, 50%, and 75% were defined as having a low, moderate, and high level of heterogeneity, respectively [32]. An alpha level of 0.05 was defined for the statistical significance of all the tests.

### Risk of Bias Assessment and Methodological Quality

To assess the methodological quality of the included studies, the PEDro scale was used. Two independent researchers assessed 11 methodological issues by assigning either one or no point. Note that studies with a higher score represent a higher methodological quality. If any conflict between the ratings of the two researchers was found, the methodological issues were reassessed and discussed. Moreover, to assess a possible publication bias, visual inspection of the funnel plot and the statistics of the Egger’s regression intercept test were used.

## Results

### Results of the Search

**Fig. 1:**
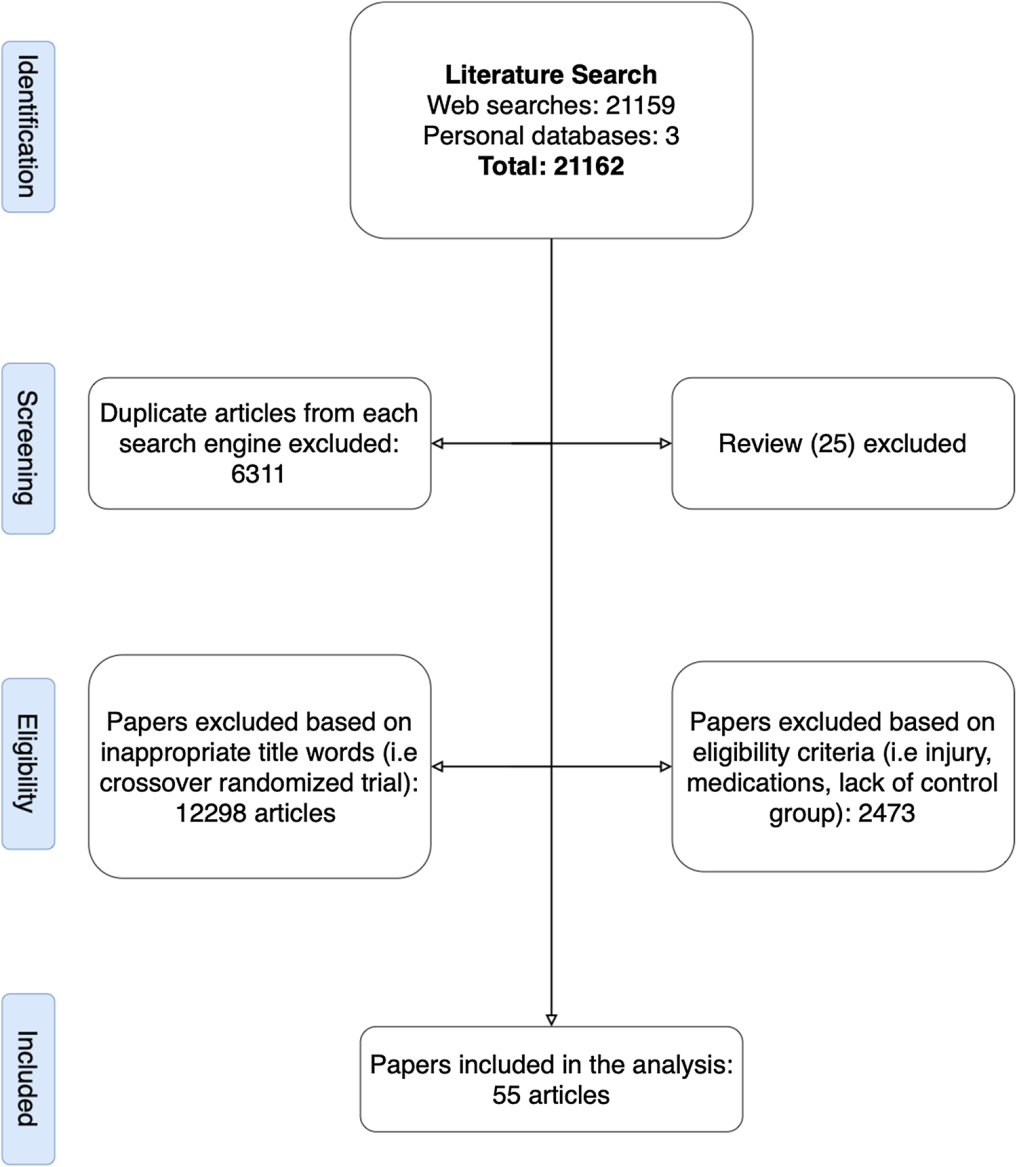
Preferred Reporting Items for Systematic reviews and Meta-Analyses (PRISMA) flow chart illustrating different phases of the search and study selection

Overall, after removal of the duplicates, 14,851 papers were screened, from which 52 papers were found to be eligible for this review. However, following the additional search of the references (search through the reference list) and citations (search through Google Scholar) of the 52 already included papers, three more papers were identified as relevant. Therefore, in total, 55 papers were included in this systematic review and meta-analysis. The search process is illustrated in Fig. 1. Overall, 222 ESs could be extracted from these studies. In summary, 2756 participants with a mean age of 23.9 ± 6.3 years (range 8.1–78.8 years) participated in the included studies. The characteristics and outcomes of the 55 studies are provided in Table S1 (characteristics table) of the Electronic Supplementary Material (ESM).

### Risk of Bias Assessment and Methodological Quality

**Fig. 2:**
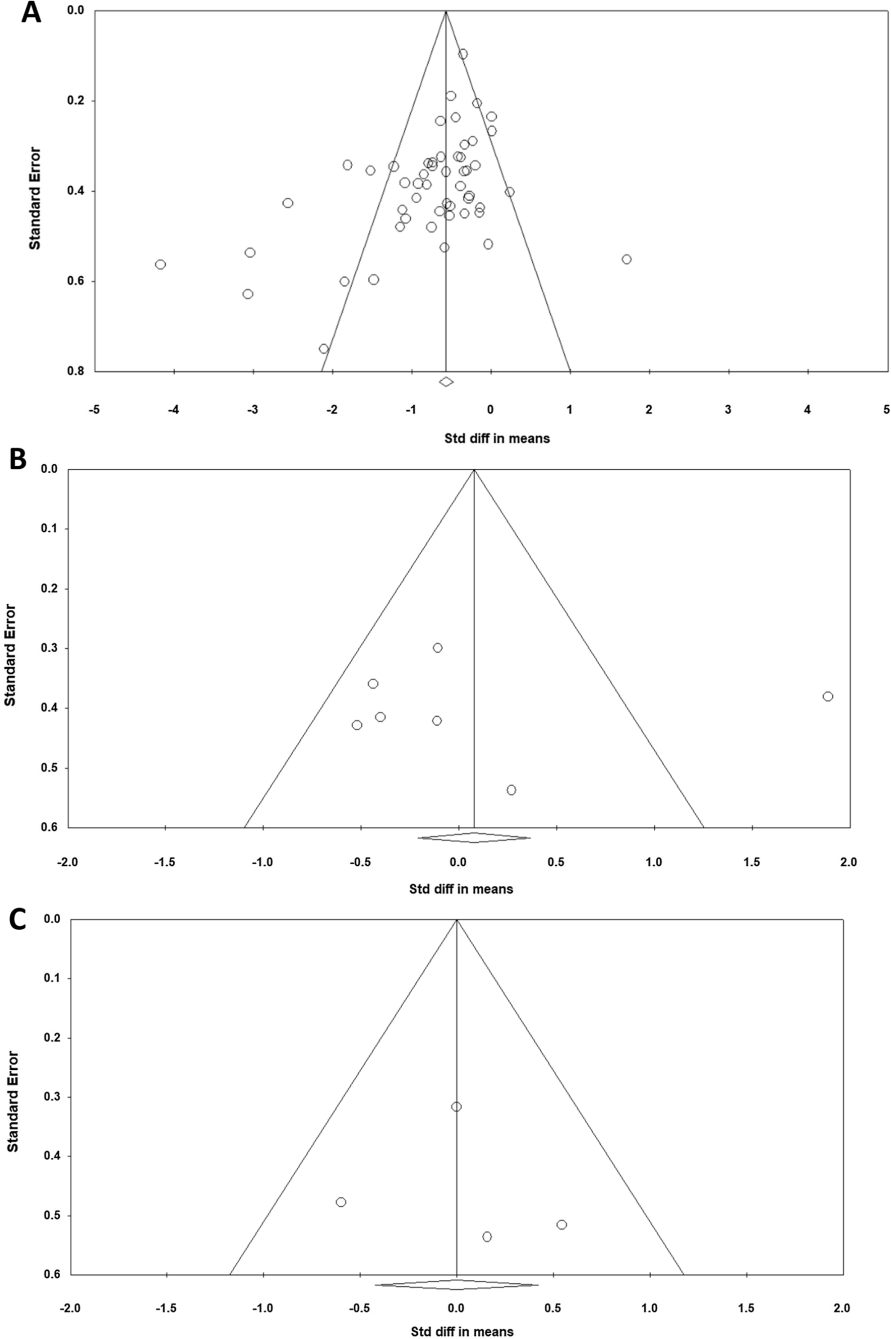
Funnel plot analysis; **A** resistance training versus controls; **B** resistance training versus stretch training; **C** resistance training and stretch training versus stretch training. *Std diff* standardized difference

Figure 2 shows the funnel plots of all the main meta-analyses. A visual inspection of the funnel plot and the Egger’s regression intercept test (intercept − 1.89; *p* = 0.002) indicated reporting bias for RT versus controls but not for RT versus stretch training (intercept 0.32; *p* = 0.95) and for RT and stretch training versus stretch training (intercept 0.54; *p* = 0.85). The methodological quality, as assessed with the PEDro scale, revealed a range of scores between 4 and 9 points (out of 10) for all the included studies. The average PEDro score was 6.5 (± 0.94), indicating a low risk of bias. The two assessors agreed with 96% of the 605 criteria (55 studies × 11 scores). The mismatched outcomes were discussed, and the assessors agreed on the scores presented in Table 1.

**Table 1:** PEDro scale score table

Study	EC	RD	CN	BL	SB	TB	AB	MO	IT	BG	ES	Total
Aline Barbosa et al. [33]	Y	N	N	Y	N	N	N	Y	Y	N	Y	5
Abdel-Aziem et al. [34]	Y	N	N	Y	N	N	N	Y	Y	Y	Y	6
Souza et al.[35]	Y	Y	N	Y	N	N	N	Y	Y	Y	Y	7
Cheol-Jin Kwak et al. [36]	Y	N	N	Y	N	N	N	Y	Y	Y	Y	6
Chinnavan et al. [37]	Y	Y	N	Y	N	N	N	Y	Y	N	Y	6
Christou et al. [38]	Y	N	N	Y	N	N	N	Y	Y	Y	Y	6
Da Costa et al. [39]	Y	N	N	Y	N	N	N	N	Y	Y	Y	5
Da Cruz et al. [40]	Y	Y	N	Y	N	N	N	Y	Y	Y	Y	7
de Oliveira et al. [41]	Y	Y	Y	Y	N	N	Y	Y	Y	Y	Y	9
Cyrino et al. [42]	Y	Y	N	Y	N	N	N	Y	Y	Y	Y	7
Faigenbaum et al. [23]	Y	Y	N	Y	N	N	N	Y	Y	Y	Y	7
Faigenbaum et al. [24]	Y	N	N	Y	N	N	N	Y	Y	Y	Y	6
Faigenbaum et al. [43]	Y	N	N	Y	N	N	N	Y	Y	Y	Y	6
Fatouros et al. [44]	Y	Y	N	Y	N	N	N	Y	Y	Y	Y	7
Fatouros et al. [45]	Y	Y	N	Y	N	N	N	Y	Y	Y	Y	7
Fourie et al. [46]	Y	Y	N	Y	N	N	N	Y	Y	N	Y	6
François Delvaux et al. [47]	Y	Y	Y	Y	N	N	Y	Y	Y	N	Y	8
Fritz et al. [48]	Y	Y	N	Y	N	N	N	N	Y	Y	Y	6
González-Gálvez et al. [49]	Y	Y	N	Y	N	N	N	Y	Y	Y	Y	7
Greco et al. [50]	Y	Y	N	Y	N	N	N	Y	Y	Y	Y	7
Greco et al. [51]	Y	N	N	N	N	N	N	Y	Y	Y	Y	5
Guex et al. [52]	Y	Y	N	Y	N	N	N	Y	Y	Y	Y	7
Elsangedy et al. [53]	Y	Y	N	Y	N	N	N	Y	Y	Y	Y	7
Ribeiro-Alvares et al. [54]	Y	Y	N	Y	N	N	N	Y	Y	Y	Y	7
Junior et al. [55]	Y	N	N	Y	N	N	N	Y	Y	Y	Y	6
Kalapotharakos et al. [56]	Y	Y	N	Y	N	N	N	Y	Y	Y	Y	7
Kao et al. [57]	Y	N	N	Y	N	N	N	Y	Y	Y	Y	6
Kiliç and Hinçal [58]	N	N	N	Y	N	N	N	Y	Y	N	Y	4
Kim et al. [22]	Y	Y	N	Y	N	N	N	Y	Y	Y	Y	7
Kloubec et al. [59]	N	Y	N	Y	N	N	N	Y	Y	N	Y	5
Lee et al. [60]	Y	Y	N	Y	N	N	N	Y	Y	Y	Y	7
Leite et al. [61]	Y	Y	N	Y	N	N	N	Y	Y	Y	Y	7
Kovách et al. [62]	Y	Y	N	Y	N	N	N	Y	Y	Y	Y	7
Manshouri et al. [63]	N	Y	N	Y	N	N	N	Y	Y	N	N	4
Monteiro et al. [64]	Y	Y	N	Y	N	N	N	Y	Y	Y	Y	7
Moraes et al. [26]	Y	Y	N	Y	N	N	N	Y	Y	Y	Y	7
Morton et al. [20]	N	Y	N	Y	N	N	N	Y	Y	Y	Y	6
Mueller et al. [65]	Y	Y	N	Y	N	N	N	Y	Y	Y	Y	7
Nelson and Bandy [19]	Y	Y	N	Y	N	N	N	Y	Y	Y	Y	7
Nobuo Takeshima et al. [66]	Y	N	N	Y	N	N	N	Y	Y	Y	Y	6
Ruslan et al. [67]	Y	Y	N	Y	N	N	N	Y	Y	Y	Y	7
Potier et al. [68]	Y	Y	N	Y	N	N	N	Y	Y	N	Y	6
Rayes et al. [69]	Y	Y	Y	Y	N	N	N	N	Y	Y	Y	7
Reinold et al. [70]	Y	Y	N	Y	N	N	N	Y	Y	Y	Y	7
Sima˜O et al. [25]	Y	Y	N	Y	N	N	N	Y	Y	Y	Y	7
Rok Vatovec et al. [71]	Y	Y	N	Y	N	N	N	Y	Y	Y	Y	7
Santos et al. [72]	Y	N	N	Y	N	N	N	Y	Y	Y	Y	6
Saraiva et al. [73]	Y	N	N	Y	N	N	N	Y	Y	Y	Y	6
Sekendiz et al. [74]	Y	Y	N	Y	N	N	N	Y	Y	Y	Y	7
Sinđić et al. [75]	Y	Y	N	Y	N	N	N	Y	Y	Y	Y	7
Phrompaet et al. [76]	Y	Y	Y	Y	N	N	Y	Y	Y	Y	Y	9
Swank et al. [77]	Y	N	N	N	N	N	N	Y	Y	Y	Y	5
Versic et al. [78]	Y	N	N	Y	N	N	N	Y	Y	Y	Y	6
Wyon et al. [21]	N	Y	Y	Y	N	N	N	Y	Y	Y	Y	7
Yaprak et al. [79]	Y	Y	N	Y	N	N	N	Y	Y	Y	Y	7
Mean												6.5
Median												7
Mode												7

PEDro scale criteria: *AB* there was blinding of all assessors who measured at least one key outcome, *BG* the results of between-group statistical comparisons were reported for at least one key outcome, *BL* the groups were similar at baseline regarding the most important prognostic indicators, *CS* allocation was concealed, *EC* eligibility criteria were specified, *ES* the study provided both point measures and measures of variability for at least one key outcome, *IT* all subjects for whom outcome measures were available received the treatment or control condition as allocated or, where this was not the case, data for at least one key outcome were analyzed by “intention to treat”, *MO* measures of at least one key outcome were obtained from more than 85% of the subjects initially allocated to groups, *N* no, *RD* subjects were randomly allocated to groups (in a crossover study, subjects were randomly allocated an order in which treatments were received), *SB* there was blinding of all subjects, *TB* there was blinding of all therapists/researchers who administered the therapy/protocol, *Y* yes

### Main Analysis

**Fig. 3:**
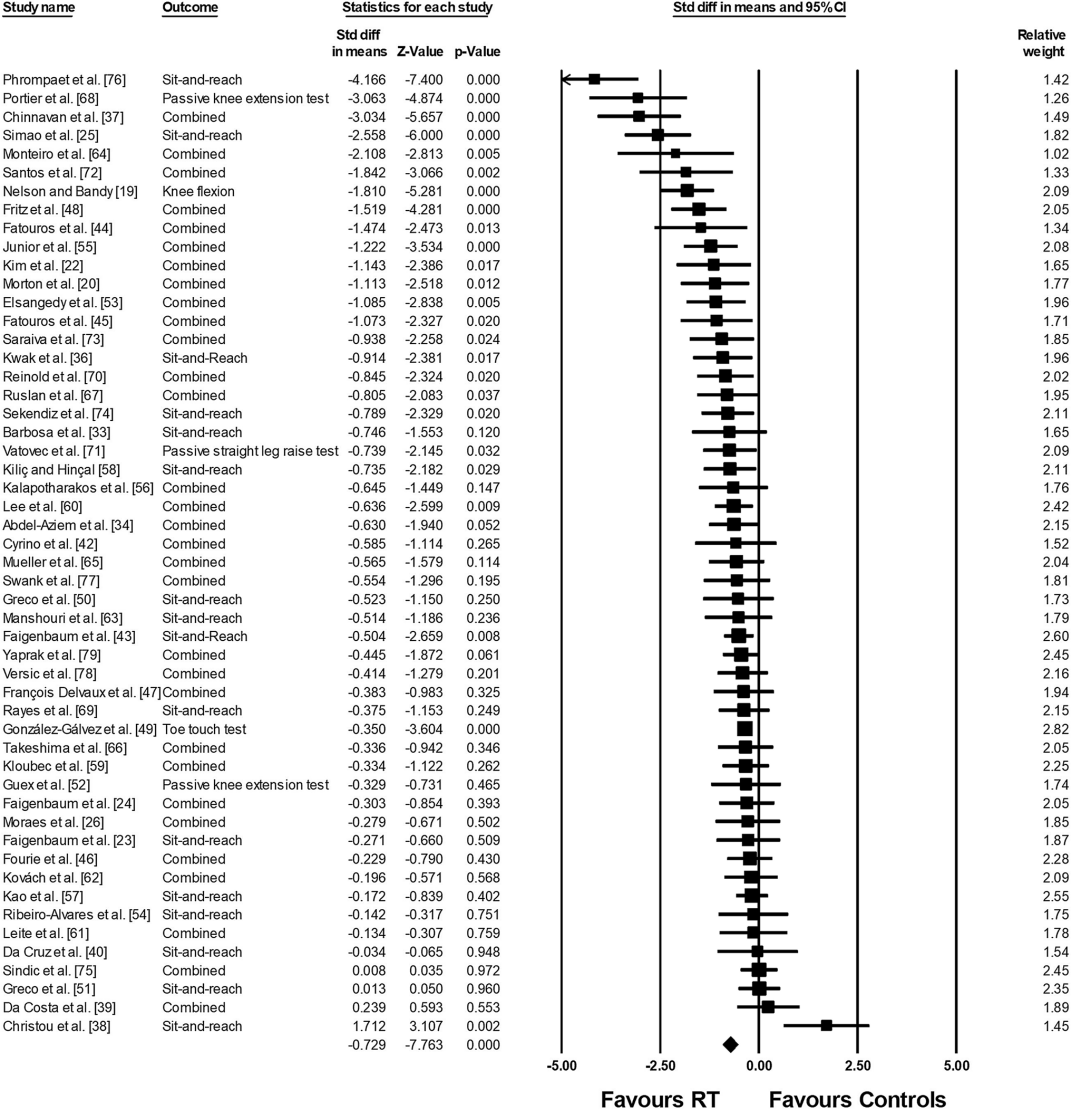
Forest plot presenting the 52 included studies with 183 effect sizes investigating the effects of resistance training (RT) on range of motion. *CI* confidence interval, *Std diff* standardized difference

**Fig. 4:**
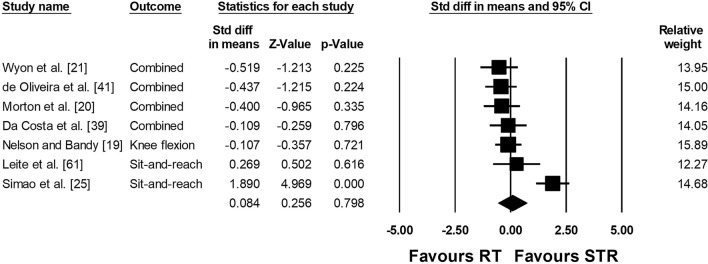
Forest plot presenting the seven included studies with 23 effect sizes comparing the effects of resistance training (RT) and stretch training (STR) on range of motion. *CI* confidence interval, *Std diff* standardized difference

**Fig. 5:**
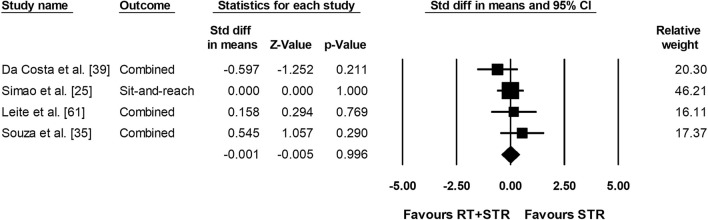
Forest plot presenting the four included studies with 14 effect sizes comparing the effects of resistance training (RT) including stretch training (STR) versus STR alone on range of motion. *CI* confidence interval, *Std diff* standardized difference

The meta-analysis on joint ROM revealed a moderate ES in favor of RT compared with the control condition (ES = − 0.729; *Z* = − 7.763; 95% confidence interval − 0.913 to − 0.545; *p* < 0.001; *I*^2^ = 73.76; number of ES = 183; number of studies = 52). Figure 3 presents the forest plot of the meta-analysis, sorted by the standard difference in means beginning with the lowest value (− 4.166: [76]) up to the highest value (1.712: [38]). Figures 4 and 5 illustrate the lack of difference in ROM improvements when comparing RT to stretch training (Fig. 4: ES = 0.084; *Z* = 0.256; 95% confidence interval − 0.558 to 0.725; *p* = 0.79; *I*^2^ = 79.12; number of ES = 23; number of studies = 7) and stretch training to a combination of RT and stretch training (Fig. 5 ES =  − 0.001; *Z* =  − 0.005; 95% confidence interval − 0.422 to 0.420; *p* = 0.996; *I*^2^ = 0.00; number of ES = 14; number of studies = 4).

A sensitivity analysis indicated that by removing the lowest ES (− 4.166 by Phrompaet et al. [76]), there was still a significant moderate effect in favor of RT whilst heterogeneity decreased slightly (ES = − 0.669; *p* < 0.001, *I*^2^ = 67.37%). Similarly, the removal of the highest ES (1.712 by Christou et al. [38]) still provided a significant moderate effect in favor of RT with a similar heterogeneity (ES = − 0.759; *p* < 0.001, *I*^2^ = 71.78%). Finally, by removing both the highest and lowest ES [38, 76], we also found a significant moderate effect in favor of RT while heterogeneity decreased (ES = − 0.696; *p* < 0.001, *I*^2^ = 64.07%).

### Moderating Variables

A summary of the subgroup analyses is provided in Table 2. Q statistics of the subgroup analyses revealed no significant differences between sex (male vs female vs mixed), the type of contraction (concentric vs eccentric), and the joint tested (elbow vs hip vs knee vs shoulder vs trunk vs trunk and hip). However, a further subgroup analysis showed a significant (*p* = 0.005) and greater magnitude of change with the increases in ROM for “untrained and sedentary” (ES = 1.042; *p* < 0.001) compared with “trained or active people” (ES = 0.43; *p* < 0.001). Furthermore, Q statistics revealed a significant difference between the types of exercise (*p* = 0.02). While RT with resistance bands, free weights, machines, Pilates, or mixed exercises (e.g., combination of free weights and machines) showed an increase in ROM, body weight exercises showed no such change (*p* = 0.11). Meta-regression showed no significant 2*p* > 0.05) between the ESs to age (*R*^2^ = − 0.07), weeks of RT intervention (*R*^2^ = 0.02), or training sessions per week (*R*^2^ = − 0.01), respectively.

**Table 2:** Statistics of the subgroup analyses

Subgroup	Number of measures	Std diff in means (95% CI)		*p* value	Q statistics
Sex					
Male	12	− 0.601	(− 1.055 to − 0.147)	0.009*	
Female	19	− 0.778	(− 1.047 to − 0.509)	< 0.001*	
Mixed	21	− 0.763	(− 1.081 to − 0.444)	< 0.001*	
*Overall*	*52*	− *0.742*	*(*− *0.930 to* − *0.555)*	< 0.001*	(*Q* = 0.455; *df* (*Q*) = 2; *p* = 0.796)
Trained state					
Trained and active	18	− 0.434	(− 0.655 to − 0.214)	< 0.001*	
Untrained and sedentary	22	− 1.027	(− 1.374 to − 0.680)	< 0.001*	
*Overall*	*40*	− *0.604*	*(*− *0.790 to* − *0.418)*	< 0.001*	(*Q* = 7.974; df (Q) = 1; *p* = 0.005)^#^
Type of contraction					
Eccentric	9	− 0.889	(− 1.376 to − 0.402)	< 0.001*	
Concentric	42	− 0.715	(− 0.924 to − 0.507)	< 0.001*	
*Overall*	*51*	− *0.742*	*(*− *0.934 to* − *0.551)*	< 0.001*	(*Q* = 0.411; *df* (*Q*) = 1; *p* = 0.521)
Type of exercise					
Body weight	4	− 0.232	(− 0.517 to 0.053)	0.111	
Free weight	3	− 1.312	(− 2.494 to − 0.130)	0.03*	
Machine	12	− 0.957	(− 1.343 to − 0.572)	< 0.001*	
Mixed	12	− 0.523	(− 0.926 to − 0.119)	0.011*	
Pilates	16	− 0.695	(− 1.034 to − 0.356)	< 0.001*	
Resistance bands	4	− 1.125	(− 1.793 to -0.457)	0.001*	
*Overall*	*51*	− *0.598*	*(*− *0.762 to* − *0.433)*	< 0.001*	(*Q* = 13.4917; *df* (*Q*) = 5; *p* = 0.016)^#^
Joint tested					
Elbow	5	− 0.530	(− 0.921 to − 0.138)	0.008*	
Hip	13	− 0.685	(− 0.899 to − 0.471)	< 0.001*	
Knee	13	− 1.021	(− 1.475 to − 0.567)	< 0.001*	
Shoulder	15	− 0.706	(− 1.071 to − 0.341)	< 0.001*	
Trunk	5	− 1.923	(− 3.222 to − 0.624)	0.004*	
Trunk and hip	34	− 0.669	(− 0.901 to − 0.437)	< 0.001*	
*Overall*	*85*	− *0.705*	*(*− *0.835 to* − *0.576)*	< 0.001*	(*Q* = 6.136; *df* (*Q*) = 5; *p* = 0.293)

*CI* confidence interval, *Std diff* standardized difference, * significant difference within a group, # significant difference between groups

## Discussion

The major findings of this meta-analysis were that RT (free weights, machines, Pilates) significantly improves joint ROM (ES = 0.73; *p* < 0.001) with the exception of no significant ROM improvement with RT using body mass. Furthermore, the beneficial effects of RT on ROM were not significantly different from stretch training or the combination of RT and stretch training versus stretch training alone. Although both groups improved ROM with RT, “untrained and sedentary” individuals had a significantly (*p* = 0.005) higher magnitude of ROM change (ES = 1.042; *p* < 0.001) compared with “trained or active people” (ES = 0.43; *p* < 0.001). There were no significant differences between sex or contraction type (e.g., concentric vs eccentric). A meta-regression showed no effect of age, training duration, or frequency.

A Current Opinion article by Nuzzo [5] based on 15 publications (nine articles measured sit and reach, six articles measured ROM with tests other than sit and reach) led him to propose that stretching does not need to be a standard component of exercise because RT and other activities are sufficient for promoting flexibility increases. Nuzzo reported individual study percentage changes in ROM with RT but did not provide information on overall mean changes, ESs (magnitude of change that incorporates standard deviation), or a full meta-analysis (includes ESs as well as measures of sensitivity, bias, and heterogeneity). Subsequently, Afonso et al. [12] did perform a meta-analysis based on 11 studies and reported no significant differences between RT and stretch training for improving ROM. However, they did observe a small ES in favor of stretching (Hedges’g = − 0.22; *p* = 0.21) over RT for improving ROM. The current meta-analysis generally concurs with the findings of Nuzzo [5] and Afonso et al. [12] based on an expanded analysis of 55 studies. Compared with controls, RT induced a moderate magnitude (standard difference in means: 0.73) increase in ROM, which was not significantly different from the increases incurred with stretch training or a combination of RT and stretch training. The current meta-analysis delved deeper than the prior reviews by differentiating between free weight, machine, body mass, and Pilates RT. Interestingly, only RT with body mass did not provide a significant increase in ROM. However, caution should be taken not to overemphasize these results, as this finding is based on only four ESs (see Table 2).

When contemplating the mechanics of free weight, machine, and Pilates RT on ROM, one might propose that the actions are similar to dynamic stretching albeit with an additional external load. Dynamic stretching has been described as an action that involves controlled movement through the active joint ROM [3, 8, 80]. Free weights and machines (including Pilates) RT typically permits the joints to reach their endpoint ROM or the individual’s point of maximum discomfort at a controlled pace. In contrast, RT with body mass activities may not always permit such an expansive ROM. For example, while a push-up is restricted by chest circumference and the surface (i.e., floor or ground), free weights and machines can permit the shoulder to surpass this more restricted ROM. There are a dearth of studies comparing the effects of partial versus full ROM RT on ROM. Kawama et al. [81] reported significantly greater decreases in the shear modulus of the semimembranosus with eccentric RT through a wide ROM versus either eccentric RT through a narrow ROM or concentric RT with a wide ROM. Furthermore, dynamic stretching involves repeated cyclical muscle loading and unloading [80]. The addition of an external load with RT would augment the stress on musculotendinous and connective tissue and minimize the unloading component. As there are no studies comparing dynamic stretch training and RT on ROM, possible differential effects of these two activities on ROM should be investigated.

Dynamic stretching in some studies has been reported to produce similar [82, 83] as well as greater [84, 85] acute increases in ROM when compared to static stretching. While one study reported more than double the ROM improvements with static versus dynamic stretch training [86], another did not report any significant difference [87]. Zhou and colleagues [88], however, reported that while all dynamic stretching modes in their study improved hip extension ROM in the elderly, the greatest ROM was achieved with dynamic stretching with no additional load versus dynamic stretching with low (0.2-kg) or high (0.5-kg) loads. Hence, it is possible that the full or nearly full ROM used in isoinertial RT is more important for increasing ROM than the external load. As isoinertial RT might be described as dynamic stretching with load, the attributed mechanisms underlying ROM improvements with dynamic stretching may be similar. Generally, these stretching adaptations have been attributed to neural, morphological, and psychological adaptations.

Although all joints exhibited significant moderate-to-large magnitude ROM increases with RT, there were no significant differences in the extent of ROM improvement between the joints. This finding is partially in accord with Afonso et al. [89] who also reported significant improvements with RT in all joints analyzed; however, they did not make direct comparisons between joints. Although it is commonly known that some joints have a substantially greater ROM than others (e.g., hip flexion vs dorsiflexion) [1], the present results demonstrate that the relative RT-induced increases were similar.

In terms of possible neural adaptations, there have been reports with static stretch training (3 and 6 weeks) of reductions in tonic Ia (facilitatory) afferent feedback from muscle spindles (T-reflexes and H-reflexes), which could reduce reflex-induced contractions inducing a more relaxed muscle (disfacilitation) [90, 91]. However, dynamic stretching and isoinertial RT would tend to excite rather than disfacilitate muscle spindle activity and thus would be an unlikely chronic training-induced mechanism for increased ROM. Golgi tendon organ inhibition is more likely to occur with large amplitude stretches [92] and higher muscle tension; however, Golgi tendon organ inhibition tends to subside almost immediately (60–100 ms post-stretching) after the stimulus discontinues [93], thus it is also an unlikely candidate for chronic dynamic stretching or isoinertial RT mechanisms. Recurrent or Renshaw cell inhibition is more prevalent with acute dynamic rather than tonic contractions [94] and can induce stabilizing effects on motoneuron discharge variability, and motor unit synchronization [95]. However, there is no research to confirm whether any of these possible acute neural responses lead to chronic training adaptations.

Morphologically, there is some evidence for dynamic ballistic stretch training to decrease tendon stiffness [96]. There are also reports of acute dynamic stretch-induced decreases in passive resistive torque [10] and muscle stiffness [10, 97], suggesting a more compliant musculotendinous unit following a single session of dynamic and ballistic stretching. However, a 6-week ballistic stretch training program did not detect any significant change in muscle morphology [98]. Magnusson and colleagues [99] contend that in response to loading, tendon metabolic activity is relatively high and can undergo significant length changes allowing the tendon to adapt to changing demands (i.e., changes in tensile force, length, compliance). Furthermore, repeated loading of the tendon with stretching can shift the stress–strain curve to promote an elevated elastic modulus [100].

In contrast, a review by Thomas et al. [101] indicated that the loading of a tendon with RT increases its stiffness by modifying elastic properties versus morphological adaptations such as an increased cross-sectional area. This increase in tendon stiffness was not dependent on muscle contraction type, trained state, or age. The review also summarized that there are reports of both increases and decreases in muscle tissue stiffness with RT and thus there is a lack of clarity regarding the effects of RT on muscle stiffness. Hence, it is unknown whether the increases and possible increases in tendon and muscle stiffness, respectively, with RT are counterbalanced by increased compliance with dynamic stretching.

Eccentric actions demand that the muscle produce force at extended positions and thus might be expected to increase ROM [12]. Training studies (4–15 weeks) emphasizing either eccentric [102, 103] or concentric [103, 104] contractions have reported increases in fascicle length. However, Reeves et al. [103] reported significantly greater increases in fascicle length (20% vs 8%) and lower increases in the pennation angle (5% vs 35%) with 14 weeks (80% of 5 repetition maximum) of eccentric versus conventional (concentric and eccentric) training. A meta-analytical review of the literature demonstrated limited-to-moderate evidence that eccentric training induces significant increases in fascicle length [105]. Hence, while there is lack of clarity regarding RT-induced changes in tendon and muscle stiffness, RT may augment ROM with alterations in fascicle length and the pennation angle.

There is also strong evidence with stretching for an increase in stretch (pain) tolerance (sensory theory) [106, 107]. The discomfort associated with the external torques on the muscles and joints with isoinertial RT would contribute to this increase in pain (stretch) tolerance permitting the individual to push beyond prior limits of discomfort. Hence, the mechanisms for increasing ROM with dynamic stretching with load (isoinertial RT) would likely be related to musculotendinous unit changes in stiffness and compliance as well as augmented stretch tolerance. As there were no significant differences between sex or contraction type and the meta-regression showed no effect of age, training duration, or frequency, these reported ROM changes and mechanisms may be similar across varied populations and training parameters.

“Untrained and sedentary individuals” displayed a significantly higher magnitude of ROM change compared with “trained or active people”. This difference is likely related to the baseline level of flexibility. Trained individuals would have already experienced an increased ROM owing to the prior chronic dynamic loading on their musculotendinous units. Hence, their scope of training-induced ROM increases would be blunted compared with previously untrained individuals [1, 108]. However, the trained individuals still experienced significant ROM improvements albeit to a lesser degree than the untrained.

The funnel plot (Fig. 2A) and the Egger’s regression intercept test (intercept − 1.89; *p* = 0.002) for RT versus controls (but not for the other meta-analyses) indicated a reporting bias limitation. It is clearly established that significant positive results are more likely to be published with an increased probability that they would be published in higher impact journals and thus also achieve a higher number of citations [109, 110]. Although one must always be cautious when interpreting results, especially those with a possibility of bias, the results of the main analysis (RT vs control) of 52 studies did demonstrate moderate standardized differences in means (0.72).

As with all studies, there are limitations to this meta-analysis. The computer software program used for our analysis (Comprehensive Meta-Analysis) calculates the arithmetic mean when more than one ES from one study is taken, which does not fully take into account the dependency of ESs, and which may therefore contribute to imprecise estimates (true heterogeneity is higher). While nesting dependent ESs within each study is preferable, the Comprehensive Meta-Analysis software does not possess that capability. Both a strength and weakness of this review may be related to the fact that different ROM tests were combined in the analysis. As the meta-analysis compares standard mean differences, it allows for a comparison of disparate but related tests. However, the mean difference of changes of joints with different anatomical configurations (e.g., hip flexion and dorsiflexion ROM) or different tests for the same joint (e.g., sit and reach vs supine hip flexion) may not provide as sensitive an analysis as comparing the same joints and tests. In contrast, it does allow a comparison of a much greater volume of research articles providing a broader perspective. Regarding ecological validity, the articles in this review cover a wide spectrum of the population and training parameters. For example, the review articles include age ranges of 10–70 years, training durations of 4–24 weeks, and training frequencies of 2–5 sessions per week at 40–110% of 1RM with a spectrum of sedentary, athletic, untrained, recreationally active individuals (see Table S1 in the ESM). Finally, the inclusion of articles that the authors may be aware of in addition to those found through objective search criteria could introduce bias. However, we identified these articles and a further analysis did not detect bias.

## Conclusions

As RT with external loads can improve ROM, additional stretching prior to or after RT may not be necessary to enhance flexibility. Based on the present studies and the literature, both stretching and RT can improve ROM, improve strength [1, 111, 112], and decrease musculotendinous injury incidence [113]. When circumstances dictate (i.e., time restrictions), flexibility training benefits can be incorporated into RT; however, stretch training can still be advocated as a fitness and training component for much of the population. For example, RT would not be suitable as a component of a warm-up prior to competition and thus stretching would play an important role in certain activity or competition preparation. Stretching is also used as a form of relaxation for many practitioners, for which RT may not be as appropriate.

## Supplementary Information

Below is the link to the electronic supplementary material.

Supplementary file 1 (DOCX 31 kb)

## Data Availability

The data from articles used for this meta-analysis are available in Table S1 of the ESM.
